# Intestinal Microbiota—A Promising Target for Antiviral Therapy?

**DOI:** 10.3389/fimmu.2021.676232

**Published:** 2021-05-12

**Authors:** Mengling Yang, Yang Yang, Qingnan He, Ping Zhu, Mengqi Liu, Jiahao Xu, Mingyi Zhao

**Affiliations:** ^1^ Department of Pediatrics, The Third Xiangya Hospital, Central South University, Changsha, China; ^2^ Guangdong Cardiovascular Institute, Guangdong Provincial People’s Hospital, Guangdong Academy of Medical Sciences, Guangzhou, China

**Keywords:** COVID-19, SARS-CoV-2, intestinal microbiota, virus, immunity

## Abstract

The intestinal microbiota is thought to be an important biological barrier against enteric pathogens. Its depletion, however, also has curative effects against some viral infections, suggesting that different components of the intestinal microbiota can play both promoting and inhibitory roles depending on the type of viral infection. The two primary mechanisms by which the microbiota facilitates or inhibits viral invasion involve participation in the innate and adaptive immune responses and direct or indirect interaction with the virus, during which the abundance and composition of the intestinal microbiota might be changed by the virus. Oral administration of probiotics, faecal microbiota transplantation (FMT), and antibiotics are major therapeutic strategies for regulating intestinal microbiota balance. However, these three methods have shown limited curative effects in clinical trials. Therefore, the intestinal microbiota might represent a new and promising supplementary antiviral therapeutic target, and more efficient and safer methods for regulating the microbiota require deeper investigation. This review summarizes the latest research on the relationship among the intestinal microbiota, anti-viral immunity and viruses and the most commonly used methods for regulating the intestinal microbiota with the goal of providing new insight into the antiviral effects of the gut microbiota.

## Introduction

The intestinal microbiota has important regulatory effects on both innate and adaptive immunity. A variety of viruses have been verified in animal studies and clinical trials to disrupt the intestinal microbiota, leading to the aggravation of immune disorders, more severe symptoms and further infection (1–3). The role of the intestinal microbiota has interested scholars since the discovery of its potential links to overall health. Faced with the worldwide epidemic caused by SARS-CoV-2, *The Diagnosis and Treatment Protocol of COVID-19* (the 7th tentative version) issued by the China National Health Commission mentions that intestinal microecological regulators can prevent secondary bacterial infections by maintaining intestinal microecological balance, and it emphasizes the importance of the intestinal microbiota balance in antiviral immunity, making the intestinal microbiota a focus of anti-epidemic strategies.

Conditional pathogenic or exotic viruses in the gut inevitably encounter the intestinal microbiota during the process of invading the body, and the intestinal microbiota has been demonstrated by many experiments to promote or inhibit the invasion of specific viruses in the intestine (4). The intestinal microbiota can even affect antiviral immunity in extraintestinal organs and tissues to a certain extent (5) through the so-called “gut-lung axis” (6), “gut-liver axis” (7) and “gut-brain axis” (8). Recent studies have reported high expression of the SARS-CoV-2 receptor angiotensin-converting enzyme 2 (ACE2) on differentiated enterocytes (9) and successful viral replication in the intestinal epithelium (10), suggesting that the intestine might be another viral target organ. Consistent with these findings, clinical trials have also shown that patients with gastrointestinal symptoms have a worse prognosis (11), indicating that the intestinal microbiota may affect clinical outcomes in patients infected with SARS-CoV-2 to a certain extent by regulating the immune status of the lung or intestine and even through direct interaction with viruses.

This review summarizes the latest findings regarding the possible relationship among the intestinal microbiota, anti-viral immunity and viral infection and some of the most commonly used methods of intestinal microbiota regulation to provide a new theoretical basis and molecular strategy for controlling viral infections as well as more effective and safer methods for bacterial regulation and for identifying effective targets.

## The Regulatory Effects of Intestinal Microbiota on Immunity

Studies have shown that the intestinal microbiota plays an important role in modulating the immune system against viruses (12–15). The regulatory effects of the intestinal microbiota on viral infection are closely intertwined with local and systemic immune responses and contribute to both congenital and adaptive immune responses (16, 17). The intestinal microbiota may prevent or promote viral infections, primarily *via* bacterial components, metabolites and regulating the immune response of the host (18–20).

Short-chain fatty acids (SCFAs) are the most indispensable metabolites of the intestinal flora, including acetic acid, propionic acid, and butyric acid. SCFAs reduce the growth and adhesion of pathogenic microorganisms, improve the integrity of the epithelium, and further enhance systemic host immunity by reducing the intestinal pH, thus increasing the production of mucin (21, 22). SCFAs activate G protein-coupled receptors (GPCRs) and inhibit histone deacetylase (HDAC) to exert their biological functions (23). According to a study by Trompette A et al., SCFAs also regulate the haematopoietic function of Ly6c(-) patrolling monocytes, enhance the function of CD8 T cells, and activate GPR41 to provide protection against influenza virus infection (20). In addition to SCFAs, there are many other metabolites of the intestinal flora that are reportedly related to host immunity. Pyruvate and lactate, which are produced by the intestinal flora, help to enhance immune responses by inducing the growth of GPR31-mediated CX3CR1+ dendrites in the gut (24). Research by Steed A et al. showed that desaminotyrosine (DAT), a metabolite of the intestinal flora, protects against influenza by increasing type I IFN signalling in macrophages (25).

Toll-like receptors (TLRs) are pattern recognition receptors (PRRs). In innate immunity, TLRs recognize pathogen-associated molecular patterns (PAMPs). TLRs can recognize bacterial flagellin and single-stranded viral RNA to mediate antiviral and antibacterial immune responses (26, 27). Influenza virus infection significantly increases the mRNA expression of TLR7 in lung immune cells. Antibiotic-induced dysregulation reduces the expression of genes involved in the TLR7 signalling pathway, while probiotic intervention restores the initial expression upregulation of genes, such as TLR7 (28). Furthermore, the microbiota composition critically regulates the generation of virus-specific CD4 and CD8 T cells and antibody responses after influenza virus infection (29). The intestinal flora plays an essential role in the maintenance of immune homeostasis by strengthening the integrity of the barrier functions of the gut mucosa, which is an important aspect of systemic immunity (30, 31). Moreover, the healthy intestinal flora plays a crucial role in regulating TLR 7 signal transduction, which has been found to mitigate common mucosal immune system (MIS) damage caused by antibiotic treatment in mice (28).

In addition, many researchers are studying how the gut microbiome affects immunity in distal parts of the body, such as the lungs, brain and liver. For instance, changes in the microbial community in the lungs, including the airways, can affect the composition of the intestinal flora. In addition, some gastrointestinal diseases are also associated with alterations in the respiratory tract (32). The transduction of immunomodulatory signals and the transfer of metabolites between the lungs and gut constitute the gut-lung axis (33). The intestinal and respiratory mucous membranes provide physical barriers to microbial penetration, and the colonization of the normal microbiome is resistant to pathogens. Bacterial transfer from the gastrointestinal tract to the lungs has been observed in sepsis and acute respiratory distress syndrome, in which barrier integrity is impaired (34, 35). The gut-brain axis refers to the two-way information network between the intestinal flora and the brain. In the gut, segmented filamentous bacteria can restore the functions of B and T lymphocytes (36). T lymphocyte receptors (TLRs) are also widely distributed on neurons (37). Therefore, gut epithelial cells transport viral and bacterial metabolites to the inner environment, neurons respond to microbial components, and the nervous system interacts with these bacterial and viral components. The balance of the intestinal flora may alter the regulation of the inflammatory response and may take part in regulating emotion and behaviour (38, 39). Because the liver is exposed to gut-derived microbial metabolites and components, intestinal dysbiosis is involved in liver disease, inflammation, and fibrosis (40). The gut-liver axis is also associated with autoimmune liver diseases, such as primary biliary cholangitis (PBC) and primary sclerosing cholangitis (PSC) (41).

In conclusion, the intestinal microbiota is capable of influencing organismal immunity locally and systemically, proximally and distally. Studying the possible mechanism by which the intestinal flora regulates host immunity can provide a clearer understanding of the occurrence and development of diseases.

## Viruses Can Change the Composition of Intestinal Microbiota

Despite a lack of clinical trials, many viruses that can spread by faecal-oral transmission and primarily induce gastrointestinal symptoms have been revealed to impact the composition of the intestinal microbiota. Novel duck reovirus (NDRV), a subtype of reovirus, was shown to result in the loss of SCFA-producing bacteria and the compensatory expansion of pathogenic bacteria in poultry (42, 43). Porcine epidemic diarrhoea virus (PEDV) is another diarrhoea-related pathogen with the ability to disrupt the intestinal microbiota balance, resulting in an increased abundance of *Escherichia* and *Clostridium* (44). Changes in the intestinal microbiota induced by rotavirus are correlated with changes in physiological parameters, such as white blood cell counts and blood urea nitrogen, in neonatal calves (45). No experiments have explored the mechanisms of these changes induced by viruses. We speculate that intestinal microbiota imbalance might be a by-product of intestinal epithelial injury since these infections always cause both morphological and functional damage.

Interestingly, some viruses that are not considered to be directly involved in intestinal epithelial injury can affect the components of the intestinal microbiota.

A study of 15 COVID-19 patients in Hong Kong showed that infection with SARS-CoV-2 significantly altered the faecal microbiomes of all 15 patients, which manifested as an enrichment of opportunistic pathogens and a depletion of beneficial bacteria in patients compared to healthy individuals, and the imbalance of intestinal microbiota persisted after SARS-CoV-2 clearance (46). This finding reveals that the abundance of certain species, such as *Coprobacillus* and *Clostridium ramosum*, is correlated with COVID-19 severity (46). Consistent with the findings in Hong Kong (47), a subsequent study in which shotgun sequencing was performed on the total DNA extracted from stools from COVID-19 patients also showed a low proportion of gut microbiota with immunomodulatory potential, including *Faecalibacterium prausnitzii*, *Eubacterium rectale* and bifidobacteria. The exact mechanism by which SAR-CoV-2 infects the intestinal microbiota is not clear. COVID-19 could cause patients to experience a state of severe inflammatory stress with increased secreted proinflammatory cytokines, such as TNF-α and IL-6 (48, 49), in blood and tissues. As important mediators of inflammation in the gastrointestinal tract (50), cytokines might result in intestinal inflammation and disrupt the homeostasis of the intestinal microbiota. High expression of ACE2, the receptor of SARS-CoV-2, was recently observed in the intestinal epithelium. Although currently no evidence supports the ability of SARS-CoV-2 to invade the host through the ACE2 in the gastrointestinal tract, the possibility that SARS-CoV-2 alters the composition of the intestinal microbiota through this type of route cannot be excluded.

Other respiratory viral infections also exhibit potential for remodelling intestinal microbiota.

As early as 2014, Wang et al. (51) reported that during influenza infection, lung-derived CCR9+ CD4 T cells can be recruited to intestinal tissues and enhance the proportion of *Escherichia coli* (*E. coli*) by generating IFN-γ. Excess *E. coli* results in IL-15 overexpression, which stimulates the differentiation of CD4 T cells into Th17 cells that damage the intestine (51). Infection by respiratory syncytial virus (RSV) was also demonstrated to disrupt intestinal microbiota homeostasis (1). Recently, Groves et al. (52) found that similar changes in gut microbiota composition occur in response to RSV and influenza A virus infection and are accompanied by common symptoms, such as weight loss and inappetence. An increased ratio of *Bacteroidetes* to *Firmicutes* abundance, which is associated with reduced calorie intake (53, 54), was observed in this research as well, suggesting that inappetence might be an important cause of the changes in the gut microbiota after respiratory viral infections.

Intestinal microbiota imbalance is also common in HIV infection and is likely attributed to persistent inflammation, the direct effects of antiretroviral drugs and even HIV virions (55).

## Dual Regulatory Effect of the Intestinal Microbiota on Viral Infection

### Possible Mechanisms That Facilitate Viral Infection

**Figure 1:**
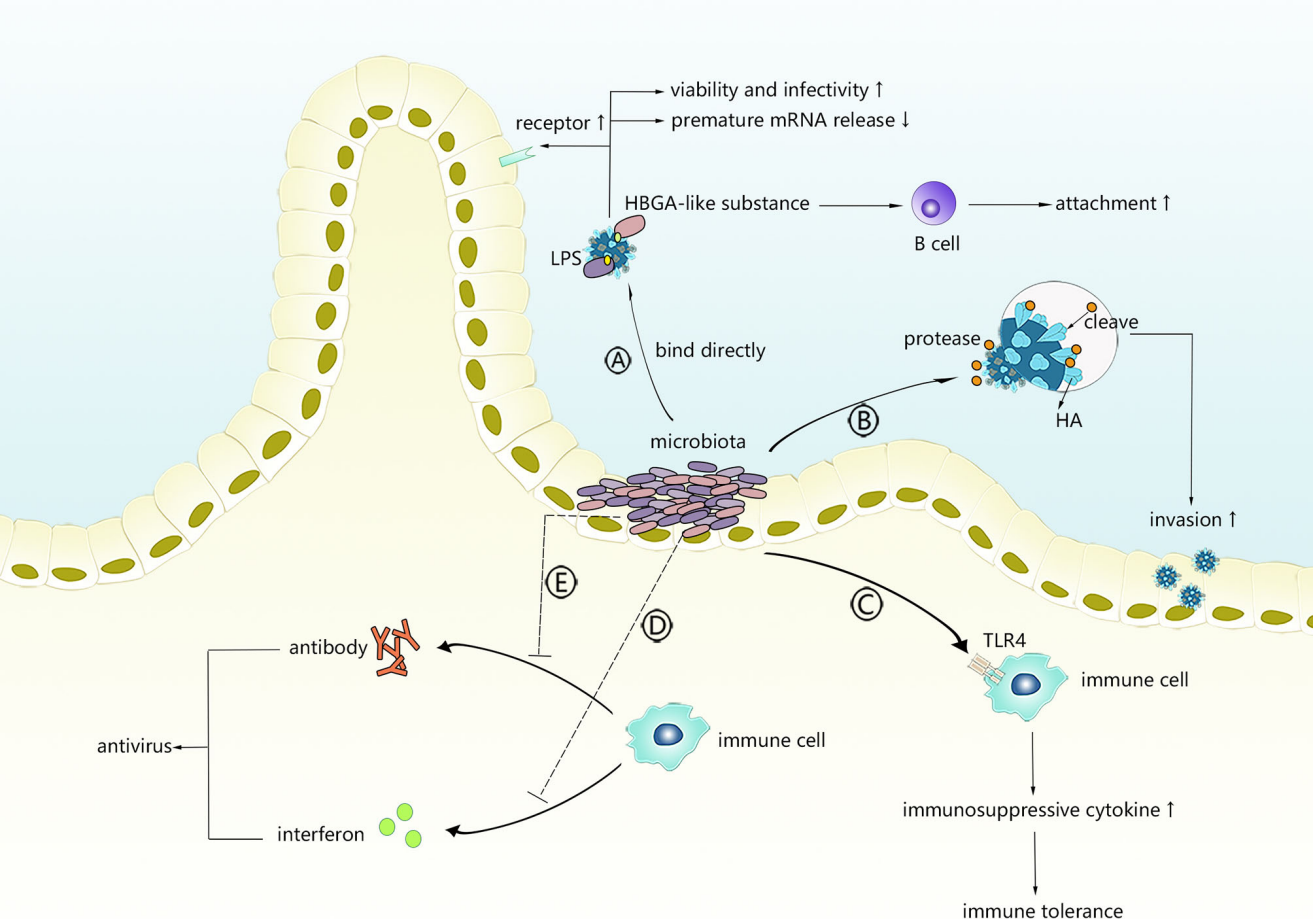
The possible mechanism of intestinal microbiota promoting virus infection. Intestinal microbiota can directly interact with viruses or regulate innate immunity or adaptive immunity. **(A)** Viruses bind with LPS or HBGA-like substances derived from intestinal microbiota. **(B)** Intestinal microbiota secretes protases to activate viruses. **(C)** Intestinal microbiota primes TLR-4 signalling to induce immunosuppressive microenvironment. **(D, E)** Intestinal microbiota interferes with production of antiviral antibodies or interferons.

As shown in **Figure 1**, intestinal microbiota might facilitate viral invasion through different mechanisms.

#### Binding to Viruses

Bacterial lipopolysaccharide (LPS), a product on the exterior surface of gram-negative bacteria, binds to and primes the signalling of its relatively specific receptor (TLR4) to initiate an appropriate or excessive immune response (56). Recently, polysaccharides, of which LPS is the most representative member, were found to bind with several enteric viruses and were positively correlated with the enhanced environmental stability of several viruses. Research conducted by Kuss et al. (57) demonstrated that poliovirus incubated with gram-negative or gram-positive bacteria exhibited significantly increased viability and infectivity, which was mediated by binding to N-acetylglucosamine (GlcNAc)-containing polysaccharides, especially LPS, and specific bacteria, such as *Bacillus*. The same results were observed in another study (58) in which LPS exposure stabilized the capsid against chlorine bleach at high temperatures and delayed its RNA release, suggesting that binding to LPS might stabilize the particles by limiting premature RNA release. Another result demonstrated that VP1-T99K, a mutated strain of poliovirus with a reduced LPS binding ability, showed relative instability when added to faeces (58) and verified the facilitative effects of LPS on virion stability. LPS was also shown to strengthen the binding of poliovirus to its receptor, which could partly explain why LPS enhances the attachment of poliovirus to host cells. Mammary tumour virus (MMTV) and reovirus are two additional viruses that could benefit from interacting with LPS to increase stability and attachment. Using negative stain transmission electron microscopy, Berger et al. (59) observed that the direct interaction of bacterial outer envelope components with virions mediates reovirus thermostability and infectivity, while the specific binding residues remain unclear. Findings showed that for both virions and intermediate reovirus particles (ISVP), lipoteichoic acid and N-acetylglucosamine-containing polysaccharides enhanced their thermostability, which could translate into enhanced attachment and higher infectivity instead of reovirus use of its proteinaceous cellular receptor junctional adhesion molecule-A or cell entry kinetics in a serotype-dependent manner, providing evidence that the interaction of viruses with the intestinal microbiota can aid infectious agents through enhanced biophysical properties of the virion that translates into enhanced infectivity. MMTV was demonstrated to express LPS-binding factors, such as CD14, TLR4 and MD-2, which are conducive to having LPS binding protein (LBP) on the envelope (60). Direct viral binding to LPS is thought to be the mechanism underlying facilitation in MMTV infection, in which LPS could help stabilize the virus and then prime TLR4 signalling, inducing the production of immunosuppressive cytokines that prolong the persistence of MMTV (60).

Host histo-blood group antigens (HBGAs), including ABO/H, secretor and Lewis families, are recognized as receptors by numerous viruses, including noroviruses (NoVs), rotaviruses (RVs), and coronaviruses. Some intestinal flora produce HBGA-like substances, and enteric bacteria might directly bind some viruses and affect viral invasion. Miura et al. (61) first revealed that HBGA-like substances localize on the extracellular polymeric substances (EPS) of human enteric bacteria to capture HuNoVLPs and play a key role in binding to NoVLPs. The MuNoV titre and attachment of GII.4 to intestinal B cells are reduced by the antibiotic-induced depletion of normal intestinal flora before oral infection (62). However, incubation with H-type HBGA-expressing *E. cloacae* or H antigen resulted in dose-dependent infectivity restoration, whereas neither *E. coli* without H antigen expression or LPS could rescue infectivity, confirming that HuNoV interactions with HBGA-like substances could facilitate the infection of and attachment to B cells. In another study involving microbiota-depleted mice infected with murine norovirus (MNV), faecal virus shedding was significantly decreased, while the transplantation of faeces from untreated mice restored MNV CR6 infectivity (63), suggesting that the intestinal microbiota plays an important role in persistent norovirus infection.

The results also provide insight into norovirus infection therapy, showing that molecules that possess a binding capacity to HBGA-like substances might alleviate noroviral infection by competing with virions for intestinal receptors.

However, the effects of the intestinal microbiota on viral attachment can be completely reversed by different experimental strategies. To investigate how specific bacteria influence NoV attachment to host cells, Rubio and colleagues performed competitive exclusion experiments (binding assays in the presence of bacteria), exclusion experiments (incubation with bacteria followed by incubation with P-particles) and displacement experiments (incubation with P-particles followed by incubation with bacteria) on P-particles (P-particles were structured by purified P-domains from NoV genotypes GI.1 and GII.4 and maintained their ability to bind to host receptors) and several bacteria expressing HBGA-like substances on their surface (64). In both the exclusion and displacement experiments, probiotic and non-probiotic bacteria exerted positive effects on P-particle attachment, whereas both kinds of bacteria were shown to have a negative effect on P-particle attachment in the competitive exclusion experiments. We speculate that HBGA-positive bacteria might be able to block virion attachment in suspension by reducing the available binding sites on virions, whereas other interactions, such as the binding of virions to bacteria, might lead to higher virion retention on the surface of the host cells. The regulatory effect of the intestinal microbiota on the immune microenvironment and intestinal mucosa glycosylation can also alter the stability, retention and infectivity of viruses that bind to the intestinal microbiota.

Regarding the impact on virion stability, HBGA-norovirus interactions were shown to protect noroviruses against abiotic stresses (65), but these protective effects were not observed for attachment to HBGA-positive *E. coli* or Tulane virus (66). Further studies are needed to elucidate the role of specific interactions between human norovirus and environmental matrices in virus thermal stability.

#### Secretion of Proteases

Cleavage of haemagglutinin (HA) mediated by proteases is essential for cell entry by receptor-mediated endocytosis during the process of influenza virus invasion. During the previous century, several bacteria were verified to have the ability to activate the influenza virus by cleaving HA by directly secreting or increasing the synthesis of proteases (67, 68). King et al. (69) examined isolates of the cloacal microbiota and found several protease-secreting bacteria and a variety of proteases, indicating that specific intestinal microbiota, such as *Enterococcus faecalis* and *Proteus mirabilis*, might alter the pathogenicity of influenza viruses with the help of proteases and facilitate viral invasion.

#### Induction of an Immune-Tolerant Microenvironment

PAMPs from commensal flora rather than pathogens are generally recognized by TLRs in the intestine, and the intestinal epithelium seems to tolerate the presence of commensal bacterial PAMPs, which usually do not provoke an inflammatory immune response (70). According to previous studies, the existence of intestinal commensal bacteria induces both enteric T-regs (71) and peripheral generation of Tregs (72) to limit indiscriminate inflammatory responses. Thus, viruses might take advantage of intestinal commensal bacteria by binding to their surface or products to escape the antiviral immune response. TLR4, a specific signalling receptor of gram-negative bacterial LPS, induces immune tolerance under continuous stimulation with low-dose LPS and has been shown to exert a negative effect on viral clearance and antiviral immunity in some cases.

MMTV, an enveloped retrovirus that expresses LPS-binding proteins, requires commensal and functional TLR4 bacteria to maintain persistence (73, 74). Through LPS receptors integrated in the viral envelope, such as CD14 and MD-2 (60), the virus cloaks itself in bacterial LPS and activates TLR4, leading to the production of immunosuppressive cytokines and the blockage of the antiviral response (74). MNV infection was shown to be mediated by intestinal bacteria through a similar mechanism (75). Norovirus, which does not generally cause obvious intestinal inflammation, provokes inflammatory lesions in IL10-deficient mice. The generation of inflammation requires enteric microbiota since intestinal lesions are not observed in germ-free IL10-deficient mice. Based on these findings, inducing the production of inflammatory suppressive cytokines, such as IL-10, by intestinal flora might represent a possible evasion mechanism against the antiviral immunity of MNV.

#### Interference With Interferon Production

Interferons are cytokines with critical importance to innate immune regulation in antiviral immunity, among which IFN-λ has been shown to have potent antiviral effects against multiple viruses, such as rotavirus, reovirus and norovirus. Both exogenous and endogenous IFN-λ were shown to inhibit the replication of specific viruses effectively in the intestine in animal experiments. Viral dependence on commensal bacteria and sensitivity to IFN-λ were first linked in a study on MNoV conducted by Baldridge and colleagues (63). Commensal microbiota depletion was shown to prevent persistent MNoV infection in wild-type mice, while infection was established in microbiota-deficient mice lacking Ifnlr1, Stat1 and Irf3, which are important factors for IFN-λ induction or signalling pathways, suggesting that commensal bacteria might promote the persistence of MNoV infection by decreasing antiviral responses mediated by IFN-λ (63). Through subsequent experiments, IFN-λ was revealed to have an obvious ability to establish MNoV infection independent of the adaptive immune response, which is generally thought to be required for viral clearance (76). Another interesting finding of this study is that MNoV replication was detected in haematopoietic cells, whereas IFN-λ acted on non-haematopoietic cells, suggesting that IFN-λ does not directly act on infected cells but rather exerts indirect regulatory effects. In addition to facilitating viral replication, certain enteric bacteria have the potential to promote organ damage secondary to viral infection through the IFN-λ pathways. *Helicobacter hepaticus*, which is more likely to colonize the colon under HBV infection, was found to act with some specific innate lymphoid cells (ILCs) to indirectly activate the IFN-γ/p-STAT1 axis, generating a detrimental immune microenvironment and accelerating the tumorigenesis of HCC (hepatocellular carcinoma) (77). Other enteric viruses, such as echovirus 11, enterovirus 71 and avian influenza virus, induce IFN-λ, and these findings suggest that viral infection can be indirectly controlled *via* the regulation of intestinal flora IFN-λ.

#### Interference With Antibody Production

Intestinal bacteria might reduce the immunosuppressive effects of viruses by interfering with the production of antiviral antibodies. By assessing rotavirus infection and replication and measuring the humoral responses of wild-type mice and microbiota-depleted mice in the days after rotavirus infection, Uchiyama et al. (78) demonstrated that rotavirus antibody levels in the microbiota-depleted group were significantly higher than those in the controls within the first few weeks. Although the antibody levels between the two groups were similar in the ninth week, the total IgA and IgG levels in microbiota-depleted mice were markedly higher, indicating that commensal microbiota promote RV infection by partially blocking the production of RV-specific antibodies.

Based on the fact that antibodies in acute HIV-1-infected individuals are predominantly targeted to HIV Env gp41 and cross-reactive with commensal bacteria, Trama et al. (79) hypothesized that these bacteria are ineffective in inducing antibodies against viruses, promoting the persistence of HIV infection. There is a normal subset of B cells that are reactive to intestinal commensal bacteria in memory B cell pools in the intestine. When facing HIV infection, the body might send out memory B cells that recognize the activation of the intestinal flora and control bacteria, such as *E. coli*, instead of native B cells, which induce specific HIV antibodies because the gp41 region of the HIV capsid is similar to the antigen of *E. coli*. As a result, non-neutralizing antibodies directed at Env gp41 are generated, and the restrictive effects of humoral immunity on HIV are alleviated. Intestinal commensal bacteria are very large, forming a complicated biological complex that is the source of many PAMPs, which induce a large number of memory cells that are generally tolerated by the immune system. Once viral components possess a similar conformation to bacterial antigens, specific bacteria might facilitate the virus through a similar mechanism as in HIV infection.

Possible mechanisms by which the microbiota facilitates viral infection are briefly summarized in **Table 1**.

**Table 1:** Possible mechanism by which the intestinal microbiota promotes viral infection.

Methods	Mechanisms	Viruses	Reference
**Binding to viruses**	Increasing viral stability by LPS Facilitating viral invasion by HBGA-like substances	Poliovirus Reovirus MMTV HuNoV MuNoV	(57, 58) (59) (60) (61) (63)
**Secreting proteases**	Activating viruses	Influenza virus	(67, 69)
**Inducing immune tolerance microenvironment**	Priming TLR-4 signalling Inducing production of inflammatory suppressive cytokines	MMTV MuNoV	(73, 74) (75)
**Interfering with interferon production**	Downregulating antiviral response mediated by IFN-λ Indirectly activating IFN-γ/p-STAT1 axis	MuNoV HBV	(63, 76) (77)
**Interfering with the production of antibodies**	Blocking the production of a specific antibody Inducing an invalid antibody	Rotavirus HIV	(78) (79)
**Generating metabolites**	Inducing poor CD4 T-cell reconstruction through butyrate Suppressing the expression of ISG	HIV Influenza virus	(80) (81)

### Possible Mechanisms of Viral Infection Inhibition

**Figure 2:**
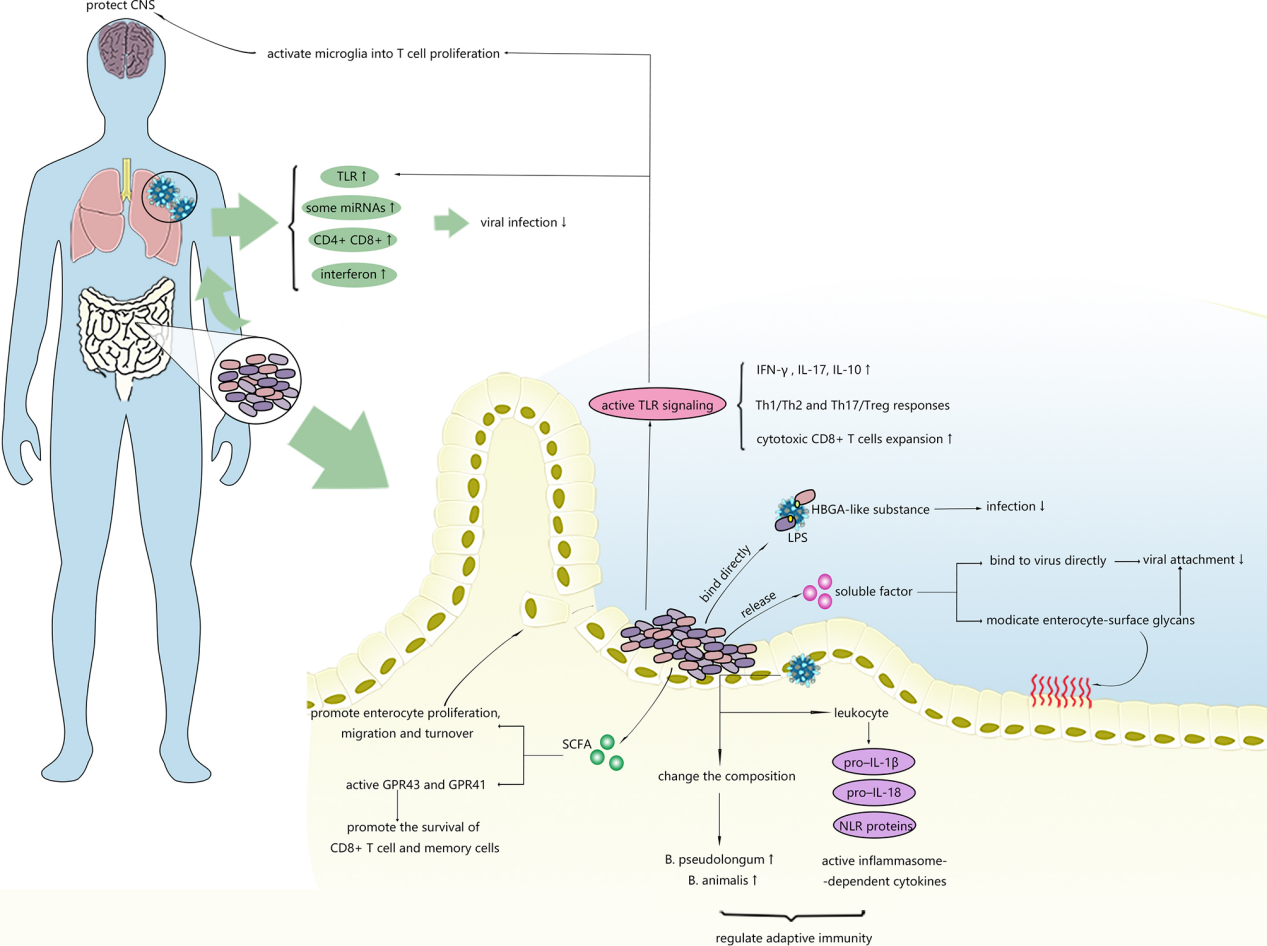
Possible mechanisms of intestinal microbiota inhibiting virus infection. Intestinal microbiota can regulate the immune response in the gut and distal tissues and affect stability of viruses.

As shown in **Figure 2**, microbiota can alleviate viral infections within and outside the intestinal tract through numerous regulatory mechanisms.

#### Stimulation of Cell Turnover

Commensal bacteria, especially gram-positive bacteria, are able to stimulate the proliferation, migration and turnover of intestinal epithelial cells (IECs) by generating SCFAs (82, 83). Recently, epithelial cell turnover induced by bacteria was revealed to potentially confer protection against certain enteric viral infections and diarrhoea. Shi et al. (84) unexpectedly discovered that the presence of unique segmented filamentous bacteria (SFB) not only protected mice from RV infections and related diarrhoea but also reduced their susceptibility to reovirus, vesicular stomatitis virus, and influenza A viral infections *in vitro*. Assessments of the transcriptional response in the intestinal epithelium showed that SFB-treated mice expressed transcripts related to pathways involved in cell turnover rather than traditional intestinal immune-mediated mechanisms, unlike untreated mice. The effects of SFB in accelerating epithelial cell turnover were also supported by observations of the increased proliferation and migration of ileum cells and the slight elongation of villi, suggesting that the stimulatory effect of commensal bacteria on host cell turnover and renewal may result in unexpected antiviral effects and might provide new targets for the treatment of RV infection or other enteric viral infections.

#### Binding to Viruses

Although most virions that bind to the intestinal microbiota exhibit increased thermal stability and stronger attachment to host cells, in some cases, the binding of viruses to the surface of bacteria might inhibit viral infection rather than promote it.

As discussed previously, LPS was previously found to bind to viruses, such as poliovirus and MMTV, resulting in increased cell attachment, persistent infectivity at elevated temperatures and increased immune evasion and transmission of the viruses (58, 60). However, influenza A virus (IAV), which is transmitted primarily *via* the faecal-oral route in wild birds, exhibits reduced stability when incubated with LPS, long-term persistence and the freeze-thaw stability of distinct HA subtypes from different host origins (85). Bandoro et al. (58) hypothesized that LPS might interact with and constrict the lipid envelope of IAV or bind to other domains of HA, except for the receptor binding site, to trigger conformational changes in HA to confer protective effects against viral fusion to host cells.

A variety of enteric bacteria have been found to express HBGA-like substances, the receptor for numerous viruses, such as norovirus and RVs. Previous studies have shown that bacteria-virus binding *via* HBGA-like substances facilitates viral invasion (62). Subsequently, relevant studies on hNoV surrogates (P particles) *in vitro* have shown that the binding of HBGAs and specific viruses serves as both an inhibitor or a promoter of viral infection in different situations (64). In pigs with HBGA-expressing *E. cloacae* colonization, which is expected to facilitate HuNoV invasion, infectivity was inhibited, and the data suggested that *E. cloacae* blocked the attachment of viral particles (86). HBGA-expressing probiotic bacteria were shown to alleviate viral infection through a similar mechanism (87). The inhibitory effects on cellular attachments of viruses *via* bacteria-virus interactions may also occur for EcN and HRV (88), since EcN was observed to primarily interact with VP4, which serves as a major viral cell attachment protein of HRV in gnotobiotic piglets. Moreover, the cellular attachment of HRV and HRV shedding were significantly reduced. The variable effects of the intestinal microbiome on viral attachment and invasion might be caused by differences in binding sites for viruses, interference with viral attachment to epithelial cells or even immunostimulatory effects on both the systemic and intestinal immune systems. The specific mechanisms by which the microbiota facilitates RV and NoV viral infection remain unclear. Furthermore, further research on whether binding to HBGA-like-coated bacteria or free HBGA-like substances aids viruses in productive attachment or stabilizes the viruses before reaching the infection sites *in vitro* and *in vivo* is needed. Experiments on P-particles and hNoV surrogates also demonstrated that specific microbiota might have the ability to inhibit hNoV invasion by binding virions in some cases.

The underlying mechanisms by which the microbiota promotes RV and NoV viral infection are far from being understood. It is also unknown whether binding to HBGA-coated bacteria or free HBGA participates in the entry process during infection, helping viruses to perform productive attachment, or just allowing the viruses to reach their infection sites.

#### Regulation of Immune-Related MicroRNAs

The beneficial effects of the intestinal microbiome on antiviral immunity are not limited to the gastrointestinal tract. Prophylactic consumption of probiotic bacteria successfully shortened the duration and reduced the severity of respiratory viral infections in clinical trials (89). It has been demonstrated that the expression of antiviral defence genes and responsive pathways in macrophages can be altered by intestinal dysbacteriosis, leading to failed control of viral infection and increased host morbidity and mortality (90). MicroRNAs (miRNAs) are considered key for interdomain molecular communication between the host and gut microorganisms. However, the role of microRNA communication in antiviral immunity regulation remains unclear. In a study conducted by Pang et al. (91), intestinal dysbacteriosis caused a decrease in miR-29c that played an antiviral role in lung tissues and led to enhanced pulmonary influenza virus amplification. Once the specific communication mechanism between the intestinal microbiota and lungs is fully understood, regulating the gut flora might be likely to help improve diseases.

#### Priming of TLR Signalling

Transmembrane cell receptors are essential for identifying pathogens in innate immunity, among which up to 10 subtypes of Toll-like receptors (TLRs) have been identified in humans. TLR7, which can recognize ssRNA from the influenza virus (92), was shown to be negatively regulated in antibiotic-treated mice after respiratory influenza viral infection, along with reduced downstream cytokines, such as IFN-γ and IL-17, and they disrupted the balance between Th1/Th2 and Th17/Treg responses (28). Immune impairment and TLR downregulation can be rescued by TLR7 ligand or the restoration of the intestinal flora, indicating that the intestinal microbiota provides protection against influenza infection by increasing the activity of the Toll-like receptor 7 (TLR7) signalling pathway. TLR7 also plays a critical role in the recognition of other viruses, such a HIV and vesicular stomatitis virus (92). Therefore, we speculate that the intestinal microbiota might also assist with viral control in infection with these viruses *via* the same mechanism.

Mounting studies have verified that clearing HBV requires mature intestinal microbiota (93–95). Evidence has revealed that exposure to low levels of microbe-derived LPS activates TLR4-mediated IL-10 secretion, eliciting liver tolerance that facilitates the persistence of HBV infection (93). Nevertheless, bacterial CpG-DNA (a TLR9 ligand) overrides liver tolerance through CpG-DNA/TLR9, increasing the expansion of HBV-specific cytotoxic CD8 T cells and leading to virus clearance (96). The prohibitive or permissive effects of the intestinal microbiota on HBV infection might depend on the strength and type of signals derived from bacteria. In addition to stimulating TLRs, the intestinal microbiota can also activate GPR43 and GPR41 by releasing SCFAs to promote the survival of CD8 T cells and memory cells (94).

The regulatory effect of the intestinal microbiota on the immune system *via* TLR signalling has recently been verified to be effective in preventing damage to the central nervous system (CNS) following viral infection. Brown et al. (97) recently demonstrated that products derived from the intestinal microbiota were sufficient to activate microglia for T cell proliferation in the CNS, providing aid against JHMV infection through microglia-intrinsic TLR4 signalling.

#### Regulation of Adaptive Immunity

The risk of respiratory syncytial virus infection is increased in infants with reduced exposure to the intestinal microbiota. Ichinohe et al. (29) revealed a correlation between influenza virulence and intestinal microbiota diversity. They noted that antibiotic-treated mice failed to mount both innate and adaptive immune responses against influenza virus infection, and this immune dysregulation was associated with the deficient generation of CD4 and CD8 T cells in lung tissue, while the local or distal injection of Toll-like receptor (TLR) ligands restored lung immunity against the influenza virus. Furthermore, loss of immunoregulation did not occur in antibiotic-treated mice infected with herpes simplex virus type 2 (HSV-2), immunity against which does not require inflammasome activation (29). These results suggest that the products of specific symbiotic bacteria trigger multiple pattern recognition receptors, stimulating leukocytes, which release factors that can support the production of pro–IL-1β, pro–IL-18, and NLR proteins, providing signalling for the activation of inflammasome-dependent cytokines. Consistent with what Ichinohe et al. (97) observed, broad-spectrum antibiotic treatment also reduced the generation of influenza-specific antibodies and T cells, weakening the ability of mice to clear the influenza virus.

Anaerobic bacteria are thought to be the primary strains modulating immune responses against influenza in the lungs by suppressing the adaptive immune response in the lungs and reducing proinflammatory cytokines, such as IFN-γ and IL-17. The anti-influenza function of anaerobes was confirmed by a recent study (98) in which a transplantation of faecal microbiota from surviving mice previously infected with virulent influenza increased the resistance of the recipient mice to influenza, indicating that the faecal material contained specific intestinal microbes with protective effects against influenza. The results further showed that the presence of *B. pseudolongum*, *Lactobacillus*, and *B. animalis* was closely correlated with survivability, and the abundances of these specific bacteria were associated with responses to influenza infection in addition to the responses of the initial gut microbes (98). Based on these findings, Zhang et al. (29) hypothesized a mechanism in which the gut microbiota might increase the abundance of endogenous *B. pseudolongum* and/or *B. animalis* to enhance the resistance of the host to influenza infection. The results of a functional metagenome analysis indicated that *B. animalis* may provide protection against influenza by promoting the biosynthesis of specific amino acids, such as valine and isoleucine, exhibiting protective effects against influenza, proposing a hypothesis regarding the mechanism underlying the protective effects of the intestinal microbiota against the influenza virus and first reporting the anti-influenza effects of *B. animalis*. Further research is needed to verify the authenticity of and specific molecular regulatory pathways involved in this hypothesis. Together, these findings suggest a protective role of intestinal bacteria in mediating the host immune response to influenza.

The above results were all derived from mouse models, and Yitbarek et al. (99) extended these findings to other species by confirming the critical role of the intestinal microbiota in controlling the H9N2 subtype of Avian Influenza Virus (AIV). As first-line innate immune factors, type I IFN levels increase after H9N2 infection in chickens, leading to the upregulation of IFN-stimulated genes and subsequent antiviral responses. The antibiotic-induced depletion of the intestinal microbiota impairs type I IFN responses in lung tissue, the gastrointestinal tract and the trachea (99). These results combined with the finding that double-stranded RNA of specific commensal intestinal microbiota has the ability to induce basal levels of type I IFNs suggest that the intestinal microbiota might initiate anti-H9N2 influenza responses *via* type-I IFN-dependent mechanisms. The expression of IL-22, which can assist in viral infection control, along with IFNs *via* IFN receptor signalling and STAT1-dependent pathways, was significantly downregulated in antibiotic-depleted chickens and was subsequently restored by treatment with probiotics or faecal microbiota transplantation (FMT), suggesting that IL-22-related mechanisms also take part in anti-H9N2 influenza immunity mediated by the intestinal flora.

The intestinal microbiota might also take part in antiviral immunity in other viral infections in addition to influenza virus infection. The antibiotic-induced depletion of the intestinal microbiota prior to LCMV infection induces physiological changes that include impaired adaptive immunity in mice, such as decreased titres of LCMV-specific IgG and the expansion of LCMV-specific CD8 T cells (90). In mice treated with oral antibiotics, susceptibility to flavivirus infections, such as severe West Nile (WNV), Dengue, and Zika virus, increases, T cell responses are impaired, and the levels of WNV-specific CD8 T cells are decreased (14). Taken together with the findings related to influenza, these results suggest that the intestinal microbiota might affect extra-gastrointestinal tract viral infections by diminishing the adaptive immune response.

#### Regulation of Glycosylation Changes on the Intestinal Surface

Numerous previous descriptive clinical studies have shown that the application of probiotics is effective at shortening the duration of viral diarrhoea or reducing rotavirus shedding (100). Soon after, their secreted soluble factors are considered effective and considerably safer for the host (101). In a study conducted by Jolly et al., RCA lectin strongly inhibited infection by both human and animal rotavirus strains in host cells (102). Subsequent studies also verified the involvement of this sugar in viral adhesion by binding with the spike protein of rotaviruses (103). In 2012, Varyukina et al. first demonstrated that bacteria-derived soluble factors that increase cell-surface galactose led to the blockage of rotavirus infections (104), indicating that modifications of intestinal epithelial cell-surface glycans caused by bacteria-derived soluble factors prevent RV attachment.

#### Antiviral Effects of Intestinal Microbiota Products

The human intestinal microbiota converts the nutrients in food into a variety of metabolites, the accumulation of which in the bloodstream regulates both local and distant immune responses. These metabolites exert metabolic and signalling functions similar to those of the metabolites of pharmaceutical agents. SCFAs, bacterial metabolites derived from the metabolism of soluble fibres by specific microbiota, were shown to improve gut homeostasis by activating GPCRs such as GPR41, GPR43, or GPR109a (105) and inhibiting histone deacetylases (106). Recently, components of SCFAs were revealed to be closely related to antiviral immunity against certain viruses. The intake of dietary fibre, which is a raw material for SCFA production in pregnant women, was demonstrated to provide protection against severe RSV in new-borns (107). Protection against rotavirus infection conferred by a high-fibre diet was also observed in animal experiments. Antunes et al. (108) demonstrated that acetate is a key protective metabolite in RV infection that helps reduce viral load and pulmonary inflammation *via* a distinct mechanism by which acetate promotes the responses of type 1 interferon and the expression of interferon-stimulated genes in lung epithelial cells by activating GPR43.

Desaminotyrosine (DAT) is another intestinal bacterial metabolite that was recently demonstrated to enhance the expression of multiple type I IFN-stimulated genes (ISGs) in the lung tissues of influenza-infected mice but conferred no benefit to animals lacking in the expression of immunity-related guanosine triphosphatase family M member 1 (Irgm1) when used alone as a treatment (25). These results combined with the findings that infection with the influenza virus results in poorer outcomes in antibiotic-treated or germ-free mice (29) suggest that certain components of the intestinal microbiota prime type I IFN affect signalling and exert distal effects on responses to influenza viruses producing DAT (25).

Dozens of metabolites may contribute to adverse complications in virus-infected patients. Some components of the intestinal microbiota, such as *Anaerococcus*, *Clostridium*, *Escherichia*, *Proteus*, *Providencia* and the *Edwardsiella* genus, help break down dietary phosphatidylcholine and are partially responsible for the production of choline, carnitine, betaine, and trimethylamine N-oxide, which are independently correlated with cardiovascular complications (109, 110). According to multiple previous studies, chronic HIV infection significantly alters the intestinal mucosa and microbiota (111), resulting in the enrichment of bacteria belonging to the genus *Prevotella* (110) that are thought to play a certain role in generating the four metabolites mentioned above. Recently, Sinha et al. (112) demonstrated that intestinal disturbances caused by HIV infection significantly enhance the levels of carnitine-related metabolites and are closely related to adverse cardiovascular events in patients. For diseases related to viral infections, studies on the correlation between gut-derived phosphatidylcholine metabolites and adverse cardiovascular complications have primarily been concentrated on HIV. Nevertheless, many other viral infections, including HCV infection (113), enhance the risk of developing coronary artery disease. Gut dysbiosis in HCV infection manifests as an acceleration of the proinflammatory microbiota (114), which might induce the production of metabolites that accelerate the development of atherosclerosis. With further study, identifying the community structure of the intestinal microbiota may be a promising method for risk assessment regarding cardiovascular diseases during viral infections.

Another product of the intestinal microbiota, butyrate, which is an SCFA, exerts contradictory effects during viral infections. Experiments have shown that butyrate contributes to the health of distant organs, such as the lungs (115). Lee et al. verified a negative correlation between butyrate-producing gut (BPG) bacteria and a risk for lower respiratory viral infections in kidney transplant recipients (116). The same results were also reported in patients undergoing allogeneic haematopoietic stem cell transplantation (117). However, for many other viral infections, BPG bacteria seem to facilitate viral infection and aggravate the development of infection. Enrichment of *F. prausnitzii*, unclassified *Subdoligranulum* sp. and *C.comes*, which have the ability to produce butyrate in HIV-1-infected individuals, is associated with poor CD4 T-cell reconstruction (18), and sodium butyrate acts as an inducing agent of Epstein-Barr virus (EBV) reactivation (80), as shown *in vitro*. Butyrate was also verified to promote cellular infection with the influenza virus, reovirus, HIV-1, human metapneumovirus, and vesicular stomatitis virus (81). Detections of related genes have shown that butyrate significantly suppresses the expression of specific antiviral IFN-stimulated genes (ISGs) by reprogramming the type I IFN-mediated innate antiviral immune response, revealing a new mechanism by which butyrate influences viral infections of cells (81).

#### Unclear Effects

Adenoviruses (AdVs) are the primary pathogens that cause severe diarrhoea in children and represent major viral pathogens in immunocompromised adults (118), which could result in intestinal microbiota imbalance *via* the disruption of epithelial cells (119). A recent study revealed that a dysfunctional intestinal microbiota could make an individual more susceptible to disease-causing AdV infections (120). However, the mechanism remains unclear.

The possible mechanisms by which the microbiota suppresses viral infection are briefly summarized in **Table 2**.

**Table 2:** Possible mechanisms by which intestinal microbiota inhibit viral infection.

Methods	Mechanisms	Viruses	Reference
**Stimulating Cell turnover**	Suppressing viral invasion	Rotavirus	(84)
**Binding to viruses**	Decreasing virus stability *in vitro* through LPS Blocking virus attachment *via* HBGA-like substances accompanied by other unclear mechanisms	Influenza virus HuNoV Rotavirus	(85) (86, 87) (88)
**Regulating the immune-related microRNAs**	Increasing miR-29c production in lung tissue	Influenza virus	(91)
**Priming TLR signalling**	Upregulating the toll-like receptor 7 (TLR7) signalling pathway Upregulating the toll-like receptor 9 (TLR9) signalling pathway Priming microglia-intrinsic TLR4 signalling	Influenza virus HBV JHMV	(28) (96) (97)
**Regulating adaptive immunity**	Increasing generation of CD4 and CD8 T cells Increasing the abundance of endogenous *B. pseudolongum* and/or *B. animalis* Upregulating IFN-stimulated genes Increasing specific CD8 T cells	Influenza virus Influenza virus Influenza virus Flavivirus	(29) (98) (99) (14)
**Regulating glycosylation changes on the intestinal surface**	Modifying epithelial cell-surface glycans through bacteria-derived soluble factors	Rotavirus	(104)
**Secreting bacterial metabolites**	Increasing interferon-stimulated gene expression by generating acetate Priming type I IFN signalling by DAT	Rotavirus Influenza virus	(108) (25)
**Unclear mechanisms**		AdV	(119, 120)

## Commonly Used Methods to Regulate the Microbiota

### Probiotics

Probiotics contain microbiota strains of lactic acid bacilli and specific non-pathogenic *E. coli* that provide beneficial properties to the host. Probiotics show great potential for treating or preventing viral-related diseases, especially respiratory virus infections and viral gastroenteritis. A new systematic review showed that ingesting probiotics improves the clinical symptoms of viral gastroenteritis, such as the duration of diarrhoea and hospitalization, suggesting that probiotics should be administered to patients with viral gastroenteritis (121). The use of probiotics is also recommended for HIV-infected patients. An increasing number of clinical trials have demonstrated that probiotics confer certain curative effects with respect to improving gastrointestinal symptoms, increasing CD4 counts and sometimes reducing the plasma HIV load in HIV(+) children, adults and even infants (122–124). Evidence-based medical research has shown that with the assistance of prebiotics, probiotics significantly increase CD4 counts, especially in females (125), which is likely a result of restoring intestinal CD4 T-cells induced by epithelial healing.

The role of probiotics in respiratory tract virus infections has been continuously examined, but high-quality evidence has not yet been produced to verify their curative effectiveness.

Previous evidence has demonstrated that probiotic administration might reduce the risk of viral upper respiratory illness, but the quality of the evidence is very low (126), and the efficacy of probiotics must be further verified. Probiotic ingestion successfully reduced the risk of influenza infection and other respiratory viral infections by 35% in people in long-term and chronic care facilities compared to the placebo group, but the results were not significant (127). In a randomized controlled trial of 152 seronegative volunteers who received a challenge from rhinovirus type 39, administering *Bifidobacterium animalis* subspecies lactis Bl-04 significantly reduced the CXCL8 response to rhinovirus infection but had no influence on subjective symptom scores, infection rate or respiratory inflammation (128). Oral ingestion of Bl-04 also appeared to interfere with rhinovirus replication, as manifested by reduced viral shedding in nasal secretions (128). These results indicate that probiotics have the potential to alter the baseline state of innate immunity and the subsequent host response to rhinovirus infection, whereas even though virus-specific CCR5+ effector memory CD4 T cells were found to be critical members in controlling rhinovirus (129), a probiotic modulation of T-cell populations or broader immune signatures in rhinovirus infection has not yet been observed. In a subsequent trial, neither rhinovirus infection nor oral probiotic consumption affected the abundance of the nasal microbiota but did influence clinical symptoms during rhinovirus infection, and the administration of probiotics through the nasal cavity might be used to treat rhinovirus-associated diseases or respiratory viral infections effectively (130).

Based on current clinical trials, we speculate that probiotic administration might be more effective in alleviating virus-related illnesses that are more likely to alter the composition of the intestinal microbiota. The species specificity of the effects of probiotics on immune function might also be an influencing factor. In conclusion, probiotics are currently recommended for treating viral gastroenteritis and HIV infection. The effects of probiotics against other viral illnesses require further verification in larger samples.

### FMT

FMT is the procedure by which microorganisms from the fresh or frozen faecal matter of healthy donors are directly transferred to a patient, and this technique has been primarily adopted for treating recurrent *Clostridium difficile* infection (131) and has been widely studied since it was approved by the U.S. Food and Drug Administration in 2003 to treat *Clostridium difficile* infection. The effects of FMT have also been shown in other gastrointestinal (GI) diseases and non-GI diseases (132).

FMT is a promising microbiota-modulating therapy for HBV- or HCV-related diseases. Administering FMT (a faecal suspension containing *Lachnospiraceae* and *Ruminococcaceae*) was observed to restore microbial diversity and function in the intestine and reduce serious adverse events in HCV-derived cirrhosis patients administered a 5-day broad-spectrum antibiotic treatment (133). A decreased ratio of *Bifidobacteriaceae*/*Enterobacteriaceae* and the translocation of intestinal bacterial products contribute to the development of HBV infection in asymptomatic carriers, chronic patients and decompensated cirrhosis patients infected with HBV (134). Therefore, FMT seems to be a promising new therapy for HBV-related illness due to its ability to reverse the proportion of certain specific bacteria in the intestine.

A recent study reported that FMT induces hepatitis B virus e-antigen (HBeAg) clearance in patients with HBeAg. In this study of 18 HBeAg-positive patients who were taking antivirals for more than 3 years, 3 out of 5 patients treated with FMT presented HBeAg clearance, while none of the 13 patients who did not receive FMT treatment exhibited HBeAg clearance (135). FMT also reduced serum HBeAg titres after each session (135). Two additional studies also verified the curative effects of FMT in clearing HBeAg; in one study, the HBsAg titres decreased after each FMT session and the serum endotoxin levels decreased (136), and in the other study, FMT resulted in a 16.7% HBeAg clearance rate in patients with chronic hepatitis B (137). In addition to having potential curative effects, FMT is a relatively simple and short-duration treatment that likely costs less than traditional repeated antiviral therapy.

Since diarrhoea induced by intestinal microbiota imbalance is an important cause of death in HIV-infected individuals, the possibility of using FMT to alleviate HIV-related illness is also of significant concern. A pilot study meant to assess the safety and efficacy of FMT in HIV infection showed that FMT application was associated with increased levels of peripheral Th17 and Th22 cells and benefitted intestinal T cell activation with no observed adverse effects (138), suggesting that FMT might represent a potential therapy for restoring T cell subset homeostasis in HIV-infected patients. However, although the latest systematic review reported that the efficacy and safety of FMT are nearly the same in patients with and without intact immunity, the safety concerns cannot be ignored since the heterogeneity of immunosuppressive subtypes makes the responses to FMT in single or combined immunocompromised states unpredictable (139).

Another issue is that the reshaping of the microbiome community structure by FMT does not last long (138), indicating that supplementary methods might be needed to maintain the remodelling of the intestinal microbiota and help with the colonization of exotic bacteria.

### Antibiotics

Antibiotics are the cornerstone of anti-infective drugs and maintain human health by targeting pathogens. Some commensal microbiota, however, might be affected more or less by antibiotic administration, especially broad-spectrum antibiotics. The overuse, prolonged use or incorrect use of antibiotics can bring up some unanticipated and undesirable consequences, including the intestinal domination of pathogenic bacteria, transient or profound loss of both microbial species and microbial diversity, increased and prolonged susceptibility to infection and the risk of reoccurring infection (140). Broad-spectrum antibiotic administration led to a significant reduction in *Bacteroidetes* and a concurrent increase in *Firmicutes*, the two groups of microbiotas that dominated over 90% of the gut communities (141). Infancy is usually considered a critical period for intestinal flora establishment due to its low diversity and the poor stability of gut microbiota compared to adults. Lu et al. found that β-lactam, a kind of antibiotic typically used in new-borns with infectious diseases, significantly reduced the overall diversity of the gut microbiota and the abundance of some beneficial bacteria, such as *Bacteroides*, in the new-borns while increasing the abundance of some opportunistic pathogenic bacteria, such as *Enterococcus* (142). Vrbanac et al. investigated the effects of ampicillin and vancomycin on the gut microbiota and metabolome and found that the local abundance of ampicillin and its metabolites was not only correlated with a loss of alpha diversity but was also related to an increased metabolome effect size. Small peptides from host proteins, including histones, were increased in the lower gastrointestinal tract of mice after treatment with these two antibiotics (143).

Fortunately, the native microbiota has a degree of self-recovery ability, and after a period of time, its composition and function will be close to those of the pre-treatment state (144). For example, frequent antibiotic administration in the NICU initially delays the maturation of the preterm neonatal microbiome, but the gut microbiota achieves a similar composition as that of antibiotic-naive term controls by 15 months of age (145). α-Defensins, the most abundant antimicrobial proteins of the intestine, are crucial for the replenishment of *Bacteroides* from the mucosal reservoir by promoting their colonization following microbiota dysbiosis induced by antibiotics (146). Although the resilience of the intestinal flora ensures that it can recover as much as possible after being disturbed by antibiotics, specific species and antibiotic-resistance genes (ARGs) still distinguish those treated with antibiotics from healthy controls (147). The abundance of ARGs increases markedly during antibiotic treatment, and the abundance of those that are chromosomally encoded decreases after antibiotic withdrawal, while the abundance of other ARGs that are episomally encoded persists for much longer periods of time (148).

There are still many cases in which a history of antibiotic therapy was more associated with the development of some diseases. A case-control study of Kawasaki disease (KD), including 50 patients and 200 control subjects, showed that the development of KD was associated with previous antibiotic administration and that antibiotics might contribute to the development of KD by affecting the intestinal microbiota in infants and young children (149). Additionally, long-term antibiotic exposure has been associated with an increased risk for several diseases, such as type 2 diabetes (150), inflammatory bowel diseases (151), and asthma (152). Therefore, the interaction of antibiotics and intestinal microbiota must be taken into account when administrating antibiotics.

### Traditional Chinese Medicine (TCM)

Given the potential risk of administering conventional medications, such as antibiotics, TCM has attracted increasing interest for many disease treatments, such as diabetes (153), ulcerative colitis (UC) (154) and kidney diseases (155), the mechanisms of which have been further demonstrated to be associated with the intestinal microbiota. The treatment of diabetes mellitus is one of the most typical examples showing that TCM’s regulatory effect on the intestinal flora exerts a therapeutic role (153, 156). Berberine (BBR), which is extracted from a traditional Chinese herb, is used to alleviate symptoms of type 2 diabetes mellitus. Yao et al. reported that the richness and diversity of gut microbiota in type 2 diabetes rats treated with BBR showed increasing trends compared to untreated rats (153). The same mechanisms were also found during the treatment of antibiotic-associated diarrhoea (AAD) with Xianglian pill (XLP), a traditional Chinese pharmaceutical preparation synthesized from BBR as a raw material (157). *Centella asiatica* (CA) is a traditional medicinal herb with a long history of anti-inflammatory application that was demonstrated to reshape the gut microbiota in UC mice by increasing the α-diversity and shifting the community by depleting colitis-associated genera to repair the intestinal mucosal barrier (154). The Baitouweng (BTW) decoction also improved inflammatory symptoms in mice with UC by modulating the intestinal microflora, including decreasing the proportion of *Firmicutes* to *Bacteroidetes* and the ratio of *Proteobacteria*, decreasing the relative abundance of *Escherichia-Shigella* and increasing the relative abundance of *Lactobacillus* and *Akkermansia* (158).

Many signalling pathways might be involved in the TCM-mediated treatment of UC through the intestinal microbiota. Kuijieyuan decoction (KD), a traditional Chinese medicine, alleviates intestinal barrier injury in ulcerative colitis, exerting antioxidant and anti-inflammatory properties by affecting TLR4-dependent PI3K/AKT/NF-κB signalling (159).

Qing et al. found that the IL-6/STAT3 pathway was suppressed by BTW treatment, resulting in a better curative effect (158). Qingchang Suppository (QCS) and its ingredients are capable of downregulating the levels of IL-6 and STAT3 in LPS-induced Caco-2 cells and of alleviating the symptoms of trinitrobenzenesulfonic acid (TNBS)-induced colitis in rats, suggesting that the JAK2/STAT3 pathway might also be a potentially involved signalling pathway. Although the exact mechanisms by which TCM improves diseases through the intestinal microbiota require further exploration, there is no doubt that TCM exerts potential therapeutic effects.

## Discussion

An increasing number of studies have indicated both direct and indirect (through the immune system) mutual regulation between the intestinal microbiota and viruses. Restoring intestinal microbiota homeostasis by using probiotics, FMT or the antibiotic-induced depletion of intestinal microbiota can affect the duration and severity of specific viral infections, as mentioned earlier. In cirrhotic patients with viral hepatitis, the restoration of microbial diversity by FMT, probiotics or prebiotics decreased the endotoxemia levels and ammonia serum and simultaneously prevented complications and improved prognoses (160). During the past decades, only limited types of antiviral drugs have been successfully developed for a few viruses, such as HIV, HSV and HCV (161). In addition, the emergence of drug-resistant viruses and the need to discover efficient targets for more kinds of viruses remain difficult problems. Under these circumstances, regulating the intestinal microbiota is a promising adjuvant therapy in viral infections. However, there are many limitations in the studies on the mechanism of mutual regulation between viruses and the intestinal microbiota. Studies on human viruses have long been hindered by the lack of a strong culture system and suitable animal models (86). For example, for HuNoV, the interaction between the same virus and the same strains of intestinal microbiota may be quite different *in vivo* and *in vitro* since other factors, such as glycosylation and mucosal immunity in the intestine, can affect viral invasion. As a result, the mechanisms that have been demonstrated in animal models may not occur in humans, and the opposite mechanisms may even occur. The regulation of the intestinal microbiota by specific viruses might be species-specific, and current regulatory methods on the intestinal microbiota in the clinic lack pertinence to some special bacteria, except for common probiotics and pathogenic bacteria and primarily work by enhancing intestinal innate immunity. In most cases, it seems that intervention measures to intestinal microbiota, including FMT or probiotic administration, restore a healthy intestinal community to improve the prognosis of viral infections, lacking discovery of the interrelationship between virions and one or several certain types of bacteria (160). Additionally, regarding FMT, ethical and social issues are present in 5 areas: (1) informed consent and the vulnerability of patients; (2) determining what a ‘suitable healthy donor’ is; (3) safety and risk; (4) commercialization and potential exploitation of vulnerable patients; and (5) public health implications (146). Although effective supervision measures are necessary, over-restriction also hinders professional care and the development of FMT.

Since the gut microbiota is a very large community that participates in mutual regulation with both the innate and adaptive immune systems of the host, a database of these complex regulations of each single intestinal microbiota type should be developed to regulate specific microbiota.

## Conclusion

This review summarizes the latest research on the relationship between the intestinal microbiota and viruses as well as the most commonly used methods for regulating the intestinal microbiota, demonstrating that the intestinal microbiota might represent a promising target for antiviral therapy. The regulation of the intestinal microbiota through probiotics and FMT is promising for viral infection therapy. However, the specific mechanisms of the interactions between the intestinal microbiota and viruses require further study, and experimental models that more closely mimic the human internal environment are needed. More evidence is needed to verify the safety and efficacy of FMT, and more targeted regulatory tools must be developed since the effects of the microbiota on viral infection depend on both the individual virus and host.

## Author Contributions

MY, YY, and MZ drafted the manuscript. YY generated the figures. ML and JX performed the background research. QH, PZ, and MZ edited the manuscript. All authors contributed to the article and approved the submitted version.

## Funding

This research was funded by the National Key Research and Development Program of China (2018YFA0108700), National Natural Science Foundation of China (81970248), and the Research Team Project of Natural Science Foundation of Guangdong Province of China (2017A03031207).

## Conflict of Interest

The authors declare that the research was conducted in the absence of any commercial or financial relationships that could be construed as a potential conflict of interest.
